# Bidirectional Predictors Between Neurobehavioural Measures During Total Sleep Deprivation and Baseline and Recovery Sleep Measures

**DOI:** 10.1111/jsr.70031

**Published:** 2025-03-08

**Authors:** Lauren N. Pasetes, Namni Goel

**Affiliations:** ^1^ Biological Rhythms Research Laboratory, Department of Psychiatry and Behavioral Sciences Rush University Medical Center Chicago Illinois USA

**Keywords:** biomarkers, neurobehavioural performance, Psychomotor Vigilance Test, recovery, sleep, sleep deprivation

## Abstract

For the first time, we examined bidirectional predictors between baseline night (B2) and recovery night 1 (R1) actigraphic sleep measures and neurobehavioural indices during total sleep deprivation (TSD) in a 5‐day experiment with 32 healthy adults. During the B2 and R1 nights, wrist actigraphy assessed sleep indices. Neurobehavioural measures were collected during B2 daytime and TSD. Simple linear regression assessed bidirectional predictors between B2 and R1 night sleep measures and TSD neurobehavioural measures. We found greater B2 sleep efficiency predicted lower TSD Karolinska Sleepiness Scale (KSS) scores, and later B2 sleep onset and sleep midpoint predicted lower TSD Profile of Mood States Fatigue (POMS‐F) scores. Overall, B2 sleep measures predicted 14.2%–17.2% of the variance in subjective sleepiness and fatigue measures during TSD. Better TSD Digit Symbol Substitution Test performance predicted shorter R1 sleep duration, longer sleep onset latency, and later sleep onset; and better TSD Digit Span Test performance predicted later sleep onset. Furthermore, greater TSD 10‐min. Psychomotor Vigilance Test (PVT) lapses predicted longer R1 sleep duration, lower sleep efficiency, greater wake after sleep onset, lower percent sleep, and later sleep offset. Overall, cognitive performance measures during TSD predicted 13.6%–29.9% of the variance in R1 sleep measures. Notably, females showed more significant predictive bidirectional relationships. Our novel findings demonstrate that baseline sleep measures predict subjective sleepiness and fatigue resilience during TSD, whereas cognitive performance resilience during TSD predicts subsequent recovery sleep measures. In summary, our results underscore predictors, mechanisms, and biomarkers between sleep health and individual differences in neurobehavioural performance during TSD.

## 1 Introduction

Adequate sleep is necessary to maintain waking cognitive performance and subjective states (Goel et al. 2013; Spaeth et al. 2012). Prior research has shown neurobehavioural decrements occur after one night of total sleep deprivation (TSD) (Yamazaki, Rosendahl‐Garcia, et al. 2022; Yamazaki, Antler, Casale, et al. 2021; Yamazaki, Antler, Lasek, et al. 2021; Moreno‐Villanueva et al. 2018; Honn et al. 2020; Lim and Dinges 2010; Wingelaar‐Jagt et al. 2024) with marked individual differences in these responses to sleep loss (Yamazaki, Antler, Casale, et al. 2021; Yamazaki, Antler, Lasek, et al. 2021; Yamazaki, Rosendahl‐Garcia, et al. 2022; Brieva et al. 2021; Casale et al. 2022; Killgore et al. 2009; Van Dongen et al. 2004; Mathew et al. 2021). Furthermore, neurobehavioural performance as well as actigraphic sleep measures show robust short‐term and long‐term trait‐like phenotypic stability across repeated exposures to baseline, TSD, and recovery conditions (Dennis et al. 2017; Yamazaki and Goel 2020; Pasetes and Goel 2024).

Previous studies have examined relationships between sleep measures and neurobehavioural measures in conditions without sleep loss (Cosgrave et al. 2018; Gilmore et al. 2024; Leng et al. 2024; Swanson et al. 2021; Neylan et al. 2010). Only two studies have examined whether sleep measures derived from polysomnography or electroencephalography predicted neurobehavioural measures during sleep restriction (Mao et al. 2023; Jones et al. 2022), but no prior studies have examined whether baseline actigraphic sleep measures the night before sleep loss are acute predictors of neurobehavioural metrics during TSD. Furthermore, no research has assessed whether neurobehavioural metrics during TSD are acute predictors of subsequent recovery sleep measures.

For the first time, we examined whether baseline actigraphic sleep measures the night before TSD predicted cognitive and subjective measures during TSD in healthy adults under controlled conditions. In addition, this is the first assessment of whether cognitive and subjective measures during TSD predicted sleep measures during that night's recovery. We hypothesised that (1) better sleep health metrics the night before TSD would significantly predict less subjective sleepiness and fatigue and better cognitive performance during TSD (more resilience); and (2) less subjective sleepiness and fatigue and better cognitive performance during TSD (more resilience) would significantly predict better subsequent recovery sleep health measures.

## 2 Materials and Methods

### 2.1 Participants

The Human Research Program Human Exploration Research Analog (HERA) is an isolated, high‐fidelity space analog facility located in Johnson Space Center in Houston, TX, USA. We studied 32 healthy adults in this highly controlled facility (ages 27–53; mean age ± standard deviation [SD], 35.1 ± 7.1 years, 14 females: 35.8 ± 7.6 years; 18 males: 34.6 ± 7.0 years), in which four participants at a time took part in one of four HERA 14‐day studies or one of four HERA 30‐day studies. Participants had technical and/or scientific backgrounds relevant for space exploration and human‐support skills. Participants were extensively screened by the National Aeronautics and Space Administration (NASA). Inclusion/exclusion criteria included both females and males; all participants were in excellent health—they passed a psychological assessment, a physical exam, and a drug screen, and had no history of neurological, integumentary, gastrointestinal, musculoskeletal, or cardiovascular problems (Yamazaki, Antler, Casale, et al. 2021; Yamazaki, Rosendahl‐Garcia, et al. 2022; Pasetes et al. 2023a, 2023b). Participants were not selected based on chronotype and were not screened for habitual sleep duration. The study was approved by the Institutional Review Boards of NASA, which had primary oversight, and by the University of Pennsylvania, and all protocol methods were carried out in accordance with approved guidelines and regulations. Prior to inclusion in the study, participants provided written informed consent, which was in accordance with the 1964 Helsinki Declaration and its later amendments. Participants were compensated for their participation in the protocol.

### 2.2 Procedures

During each HERA study, participants took part in a 5‐day experiment that consisted of 2 baseline nights (B1 and B2; 8‐h time‐in‐bed [TIB], 2300–0700 h), followed by 39‐h acute TSD during which participants remained awake. TSD was followed by a 10‐h TIB recovery night (R1; 2200–0800 h), and a second 8‐h TIB recovery night (R2; 2300–0700 h). Sleep and wake times were not based on chronotype, and the lights were out during bedtime hours. The 5‐day experiment occurred during study days 9–13 within the 14‐day study and during study days 23–27 within the 30‐day study. Notably, due to the homeostatic pressure to sleep following TSD, participants were allowed flexibility in bedtimes (0.5 h) and waketimes (0.5 h) for the R1 night. Fitness levels were not specifically measured; nevertheless, all participants were confined to engaging in prescribed activities at specific times, experienced similar amounts of activity during the study, and were prohibited from napping and consuming caffeine during the experiment. Participants were continuously monitored via wrist actigraphy and by outside observers to ensure adherence.

### 2.3 Actigraphy Sleep Measure Collections

A wrist accelerometer (Actiwatch Spectrum, Philips Respironics Healthcare, Bend, OR, USA) was used to measure sleep–wake metrics related to quality, timing, and quantity including sleep efficiency, wake after sleep onset, sleep onset latency, percent sleep, sleep onset, sleep offset, sleep midpoint, and sleep duration during the pre‐experimental phase (the average across 8–10 days before the start of the 5‐day experiment), and B1, B2, and R1 nights (Pasetes et al. 2023a, 2023b; Pasetes and Goel 2024). Notably, the pre‐experimental phase, and B1 and B2 nights had comparable actigraphic sleep data, with no significant differences in sleep midpoint (Table 1).

**TABLE 1:** Mean ± SD actigraphic sleep measures during the pre‐experimental phase, and baseline night 1 (B1) and baseline night 2 (B2).

Sleep measure	Pre‐experimental phase	B1	B2
Sleep duration (min.)^c^	430.515 ± 19.083	444.760 ± 28.961^b^	443.258 ± 31.620
Sleep onset (clock hour)^c^	23.607 ± 0.341	23.398 ± 0.388^a^	23.415 ± 0.534
Sleep offset (clock hour)	6.783 ± 0.133	6.820 ± 0.249^a^	6.803 ± 0.214
Sleep midpoint (clock hour)	3.195 ± 0.204	3.112 ± 0.221^a^	3.118 ± 0.311
Sleep efficiency (%)	87.552 ± 4.224	85.210 ± 16.737^a^	88.557 ± 4.711^a^
WASO (min.)	39.302 ± 17.460	37.430 ± 20.520^a^	38.161 ± 20.062
SOL (min.)	8.766 ± 7.419	11.290 ± 18.199^a^	7.678 ± 8.444^a^
Percent sleep (%)	90.877 ± 3.957	91.640 ± 4.468^a^	91.424 ± 4.248

*Note*: The pre‐experimental phase was the average across 8–10 days before the start of the 5‐day experiment. *N* = 31.

Abbreviations: B1, baseline night 1; B2, baseline night 2; SOL, sleep onset latency; WASO, wake after sleep onset.

^a^ *N* = 30.

^b^ *N* = 29.

^c^ Sleep duration [*F*(2, 56) = 5.135, *p* = 0.009] and sleep onset [*F*(2, 58) = 5.760, *p* = 0.005] demonstrated a significant effect across the pre‐experimental phase and B1 and B2 assessed by repeated measures ANOVA. Pre‐experimental phase sleep duration was significantly shorter than B2 sleep duration as assessed by post hoc analyses with Bonferroni corrections (*p* = 0.021). Pre‐experimental phase sleep onset was significantly later than B1 (*p* = 0.050) and B2 (*p* = 0.024) sleep onset as assessed by post hoc analyses with Bonferroni corrections.

Actiwatches were worn on the non‐dominant wrist, and data were collected in 1‐min. intervals (using firmware version 01.01.0015, medium wake threshold) and processed using the Actiware software (version 6.1.0). Actigraphic sleep data during the nighttime intervals were analysed similarly to our prior research (e.g., Dennis et al. 2017; Moreno‐Villanueva et al. 2018; Yamazaki, Antler, Lasek, et al. 2021; Yamazaki, Rosendahl‐Garcia, et al. 2022; Yamazaki, Casale, et al. 2022; Yamazaki and Goel 2020; Pasetes et al. 2023a, 2023b; Pasetes and Goel 2024). Notably, actigraphic data collected from past research with a similar experimental design using the same Actiwatch had sleep measures within similar ranges (Pasetes et al. 2023a, 2023b; Pasetes and Goel 2024).

### 2.4 Neurobehavioural Performance

Each participant completed precise computer‐based neurobehavioural testing sessions during the study (Yamazaki, Rosendahl‐Garcia, et al. 2022; Yamazaki, Antler, Lasek, et al. 2021). The neurobehavioural test battery (NTB) sessions were administered during B1 daytime for practice. The NTB was administered at timepoints 1130 and 1730 h during B2 daytime and then averaged for each neurobehavioural measure. The NTB was also administered at timepoints 0400, 1130, and 1730 h during TSD, and then averaged for each neurobehavioural measure (Moreno‐Villanueva et al. 2018; Yamazaki, Antler, Casale, et al. 2021; Yamazaki, Rosendahl‐Garcia, et al. 2022). The NTB included the Karolinska Sleepiness Scale (KSS) (Åkerstedt and Gillberg 1990; Yamazaki, Antler, Lasek, et al. 2021), which measures subjective sleepiness, the Profile of Mood States Fatigue scale (POMS‐F) (Bourgeois et al. 2010; Yamazaki, Antler, Lasek, et al. 2021; McNair et al. 1971), which measures subjective fatigue, and the 10‐min. Psychomotor Vigilance Test (PVT) (Basner and Dinges 2011; Yamazaki, Rosendahl‐Garcia, et al. 2022; Yamazaki, Antler, Lasek, et al. 2021), an objective behavioural attention test. The NTB also included the Digit Symbol Substitution Test (DSST) (Honn et al. 2020; Wechsler 1981; Yamazaki, Antler, Lasek, et al. 2021), which measures cognitive throughput, and the Digit Span Test (DS) (Wechsler 1981; Blackburn and Benton 1957), which measures working memory. The neurobehavioural measures analysed were KSS score, POMS‐F score, number of PVT lapses (reaction time > 500 ms), DSST # correct, and DS total # correct (sum of forward and backward # correct). The KSS, POMS‐F, PVT, DSST, and DS are highly sensitive, well‐validated measures that show robust and stable individual differences to sleep loss (Casale et al. 2022; Brieva et al. 2021; Yamazaki, Rosendahl‐Garcia, et al. 2022; Yamazaki, Casale, et al. 2022; Yamazaki, Antler, Casale, et al. 2021; Yamazaki, Antler, Lasek, et al. 2021; Basner and Dinges 2011; Åkerstedt et al. 2014; Dennis et al. 2017; Yamazaki and Goel 2020).

### 2.5 Statistical Analyses

All statistical analyses were performed using SPSS v29 (SPSS Inc., Chicago, IL, USA), with *p* < 0.05 considered statistically significant. Prior research has found normal distributions for the sleep and neurobehavioural measures examined in these studies (Yamazaki, Rosendahl‐Garcia, et al. 2022; Pasetes et al. 2023a, 2023b; Pasetes and Goel 2024; Dennis et al. 2017; Moreno‐Villanueva et al. 2018; Yamazaki and Goel 2020).

For this paper, we analysed the bidirectional predictors and relationships between B2 and R1 actigraphic sleep measures and neurobehavioural performance measures during TSD. Pearson's correlation coefficient (*r*) and Pearson's *R*‐squared (*R* ^2^ coefficient of determination) assessed relationships between B2 actigraphic sleep metrics (the last sleep opportunity prior to TSD) and TSD neurobehavioural performance measures and between TSD neurobehavioural performance measures and R1 actigraphic sleep indices. Simple linear regression analyses were conducted for significant associations determined via Pearson's correlations (Fonseca et al. 2024).

Simple linear regression analyses evaluated the extent to which each specific B2 actigraphic sleep measure (independent variable) predicted each neurobehavioural performance measure during TSD (dependent variable) and evaluated the extent to which each neurobehavioural performance measure during TSD (independent variable) predicted each R1 actigraphic sleep measure (dependent variable). Furthermore, to determine if our significant B2 actigraphic sleep results were unique predictors of TSD neurobehavioural measures, simple linear regression analyses evaluated whether B2 actigraphic sleep measures predicted neurobehavioural indices during the B2 daytime before exposure to TSD. Furthermore, we conducted multiple regression analyses with age as a factor. In addition, given the well‐established sex differences in objective sleep measures (Lok et al. 2024; Wright et al. 2023) and in cognitive performance and sleepiness and fatigue measures (Åkerstedt et al. 2017; Santhi et al. 2016), we conducted exploratory simple linear regression analyses in males and females separately on the significant main simple linear regression analyses to determine if these predictors remained significant.

Pearson's correlation coefficient (*r*) also assessed relationships between B2 sleep metrics and between R1 sleep metrics. Repeated measures (RM) ANOVA assessed differences in sleep metrics across the pre‐experimental phase and B1 and B2 nights (Table 1). Sphericity Assumed corrections for degrees of freedom were applied for all RM ANOVAs since Mauchly's test was not violated for significant within‐subject effects. Post hoc analyses with Bonferroni corrections compared the pre‐experimental phase and B1 and B2 nights when there was a significant effect across all three phases. Bonferroni‐corrected *p* values are reported in Table 1.

For *N* = 1 participant, B2 sleep onset latency and B2 sleep efficiency measures were outliers (±3 SD from the mean) and therefore were excluded from all B2 analyses. For *N* = 1 participant, R1 sleep duration, R1 sleep offset, and R1 sleep midpoint were outliers (±3 SD from the mean) and therefore were excluded from all R1 analyses (in addition, since sleep offset and sleep midpoint were both outliers, sleep onset was excluded). *N* = 1 participant was removed from all B2 and R1 analyses due to four actigraphic sleep measure outliers (±3 SD from the mean) and *N* = 1 participant did not wear the Actiwatch during R1 and was therefore excluded from all R1 analyses.

## 3 Results

### 3.1 B2 Actigraphic Sleep Measures as Predictors of Neurobehavioural Measures During TSD

**FIGURE 1:**
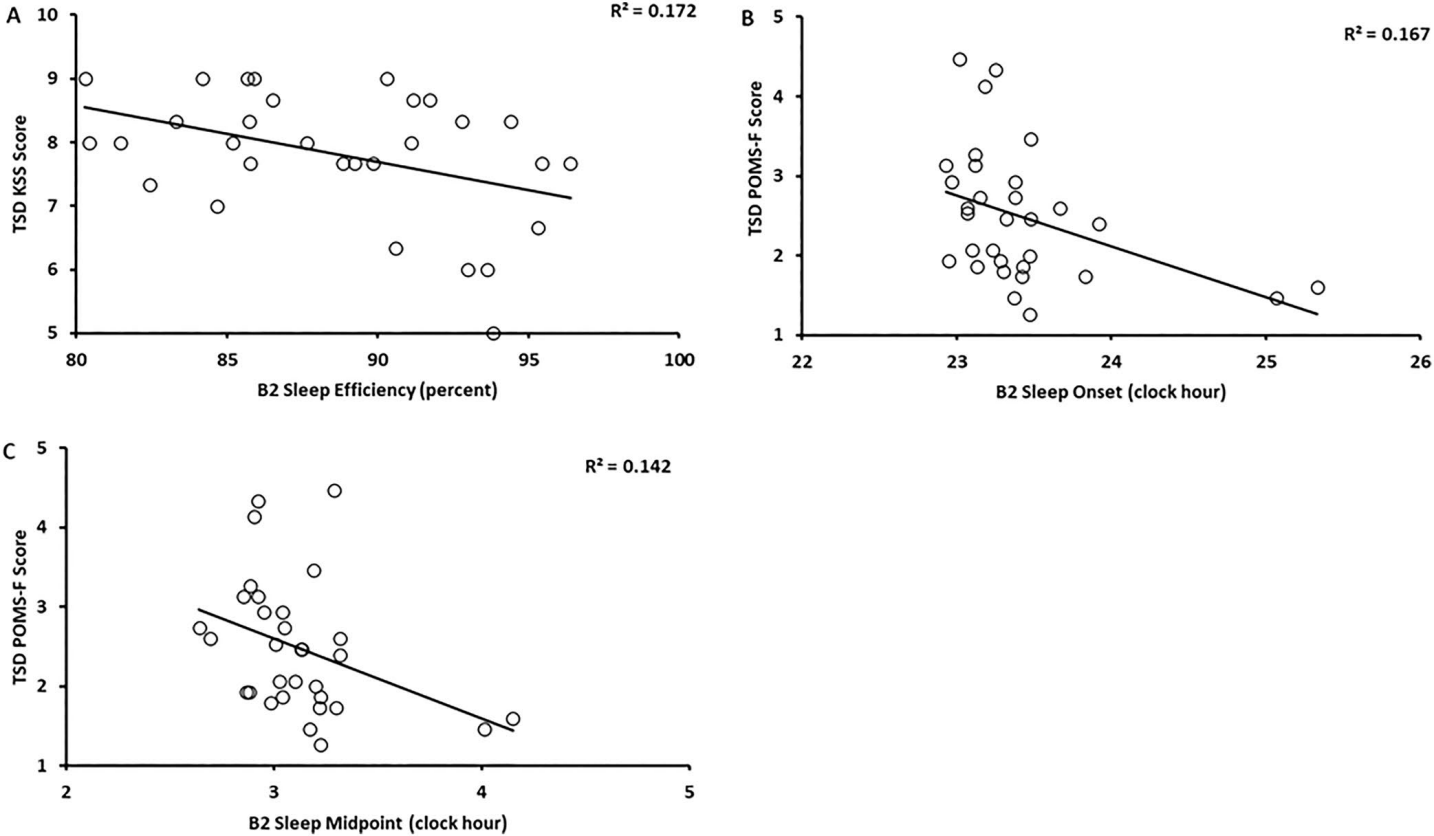
Scatter plots displaying baseline night 2 (B2) actigraphic sleep measures as significant predictors of subjective sleepiness and fatigue measures during total sleep deprivation (TSD). Simple linear regression found that (A) higher B2 sleep efficiency significantly predicted lower TSD Karolinska Sleepiness Scale (KSS) scores; (B) later B2 sleep onset and (C) later B2 sleep midpoint significantly predicted lower TSD Profile of Mood States Fatigue (POMS‐F) scores. The regression line and *R* ^2^ for each plot are shown. *p* < 0.05 was significant. *N* = 30 for (A). *N* = 31 for (B,C). For (B), on the *x*‐axis, 25 = 0100 h, 26 = 0200 h.

Simple linear regression analyses indicated that B2 actigraphic sleep measures significantly predicted subjective sleepiness and fatigue metrics during TSD. Higher B2 sleep efficiency significantly predicted less subjective sleepiness during TSD (*r* = −0.415): each 1% increase in B2 sleep efficiency significantly predicted a 0.089 decrease in TSD KSS scores (Table 2 and Figure 1A). B2 sleep efficiency predicted 17.2% of the variance in TSD KSS scores.

**TABLE 2:** Baseline night 2 (B2) actigraphic sleep metrics as significant predictors of total sleep deprivation (TSD) sleepiness and fatigue measures.

B2 sleep predictor and TSD sleepiness and fatigue measure (*N* = 31)	Unstandardized *β* ± SE	Standardised *β*	*t*	95% CI	*p* (two‐tailed)
B2 sleep efficiency and TSD KSS scores^a^	−0.089 ± 0.037	−0.415	−2.416	−0.164, −0.014	**0.022**
B2 sleep onset and TSD POMS‐F scores	−0.640 ± 0.265	−0.409	−2.411	−1.182, −0.097	**0.022**
B2 sleep midpoint and TSD POMS‐F scores	−1.010 ± 0.462	−0.376	−2.187	−1.954, −0.065	**0.037**

*Note*: Boldface indicates significant *p* values at *p* < 0.05.

Abbreviations: B2, Baseline night 2; CI, confidence interval; KSS, Karolinska Sleepiness Scale; POMS‐F, Profile of Mood States Fatigue; SE, standard error; TSD, total sleep deprivation.

^a^ *N* = 30.

In addition, later B2 sleep onset (*r* = −0.409) and later B2 sleep midpoint (*r* = −0.376) significantly predicted less subjective fatigue during TSD: each 1 h later sleep onset and each 1 h later sleep midpoint significantly predicted a 0.640–1.010 decrease in TSD POMS‐F scores, respectively (Table 2 and Figure 1B,C). B2 sleep onset predicted 16.7% of the variance in TSD POMS‐F scores, and B2 sleep midpoint predicted 14.2% of the variance in TSD POMS‐F scores. In females, each 1 h later sleep onset and each 1 h later sleep midpoint significantly predicted a 0.718 (*r* = −0.581; *R* ^2^ = 0.338) to 1.211 (*r* = −0.588; *R* ^2^ = 0.346) decrease in TSD POMS‐F scores, respectively (Table 3).

Furthermore, to determine if these significant B2 actigraphic sleep results were unique predictors of TSD neurobehavioural measures, simple linear regression analyses evaluated whether B2 actigraphic sleep measures predicted neurobehavioural indices during the B2 daytime before exposure to TSD. Higher B2 sleep efficiency significantly predicted less B2 subjective sleepiness: each 1% increase in B2 sleep efficiency significantly predicted a 0.142 decrease in B2 KSS scores (*r* = −0.396; *r* ^2^ = 0.157; *β* ± SE = −0.142 ± 0.062; *t* = −2.282; *p* = 0.030).

There were no other significant B2 actigraphic sleep measures as predictors of neurobehavioural metrics during TSD, and no other significant predictors in females and males. In addition, age was not a significant factor.

### 3.2 Cognitive Performance Measures During TSD as Predictors of R1 Actigraphic Sleep Measures

**FIGURE 2:**
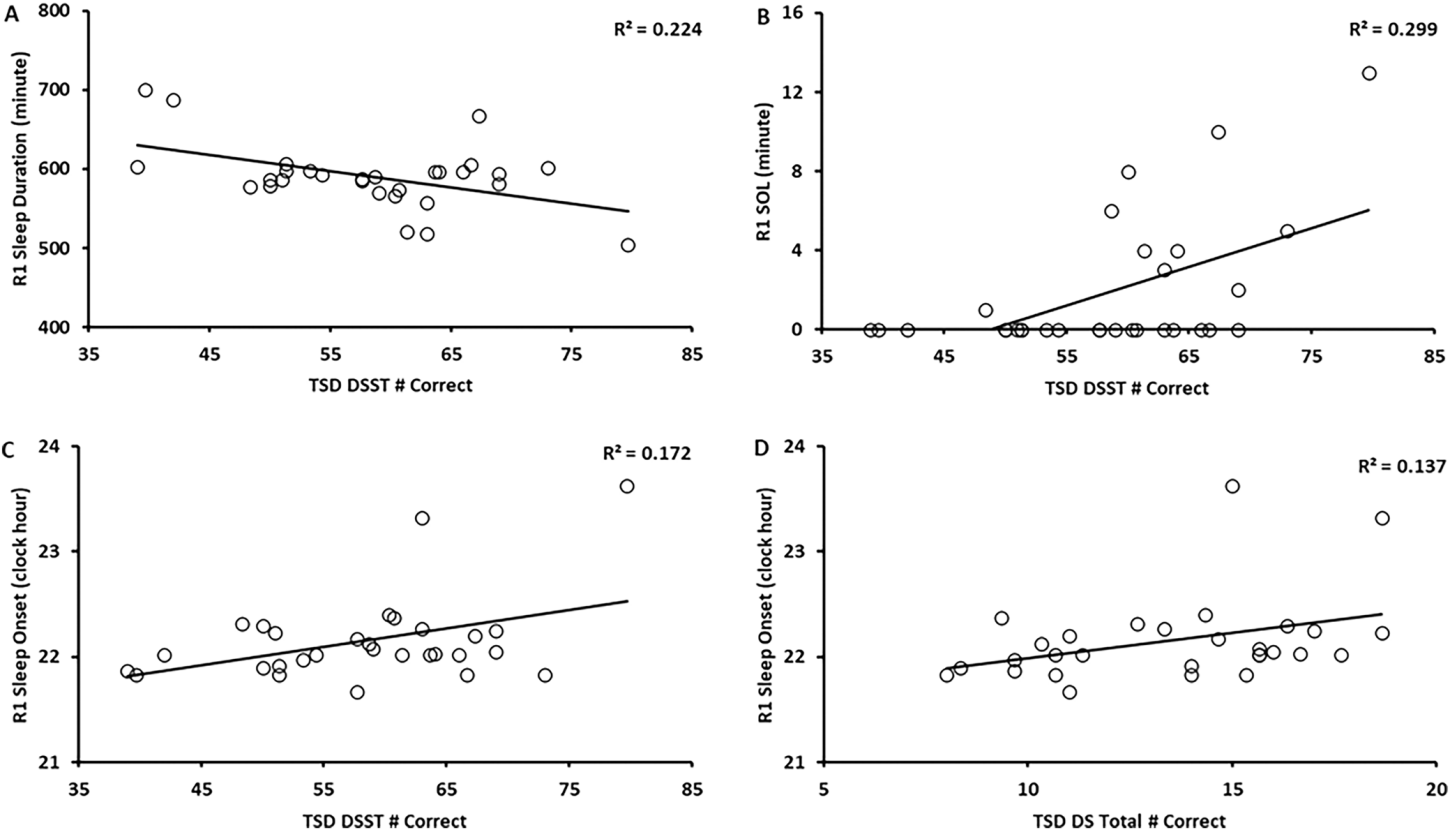
Scatter plots displaying cognitive throughput and working memory measures during total sleep deprivation (TSD) as significant predictors of recovery night 1 (R1) actigraphic sleep measures. Simple linear regression found that higher TSD Digit Symbol Substitution Test (DSST) # correct significantly predicted (A) shorter R1 sleep duration, (B) greater R1 sleep onset latency (SOL), and (C) later R1 sleep onset; and (D) higher TSD Digit Span Test (DS) total # correct significantly predicted later R1 sleep onset. The regression line and *R* ^2^ for each plot are shown. *p* < 0.05 was significant. *N* = 29 for (A), (C), and (D). *N* = 30 for (B).

Simple linear regression analyses indicated that cognitive performance measures during TSD significantly predicted R1 actigraphic sleep measures. Higher DSST # correct during TSD significantly predicted shorter R1 sleep duration, greater R1 sleep onset latency (SOL), and later R1 sleep onset (*r*: −0.473 to 0.547): each 1‐point increase in TSD DSST # correct significantly predicted a 2.029 min. decrease in R1 sleep duration, a 0.194 min. increase in R1 SOL, and a 0.017 h delay in R1 sleep onset (Table 4 and Figure 2A–C). TSD DSST # correct predicted 17.2%–29.9% of the variance in these R1 sleep measures. In females, each 1‐point increase in TSD DSST # correct significantly predicted a 2.518 min. decrease in R1 sleep duration (*r* = −0.583; *R* ^2^ = 0.340; Table 5) and in males, each 1‐point increase in TSD DSST # correct significantly predicted a 0.359 min. increase in R1 SOL (*r* = 0.683; *R* ^2^ = 0.467) and a 0.034 h delay in R1 sleep onset (*r* = 0.669; *R* ^2^ = 0.447; Table 5).

**TABLE 3:** Exploratory sex differences in baseline night 2 (B2) actigraphic sleep metrics as significant predictors of total sleep deprivation (TSD) sleepiness and fatigue measures.

B2 sleep predictor and TSD sleepiness and fatigue measure	Unstandardized *β* ± SE	Standardised *β*	*t*	95% CI	*p* (two‐tailed)
B2 sleep efficiency and TSD KSS scores					
Females	−0.044 ± 0.043	−0.291	−1.008	−0.139, 0.052	0.335
Males^a^	−0.116 ± 0.054	−0.482	−2.130	−0.232, 0.000	0.050
B2 sleep onset and TSD POMS‐F scores					
Females	−0.718 ± 0.303	−0.581	−2.369	−1.386, −0.051	**0.037**
Males	−0.549 ± 0.425	−0.307	−1.293	−1.450, 0.351	0.215
B2 sleep midpoint and TSD POMS‐F scores					
Females	−1.211 ± 0.502	−0.588	−2.411	−2.317, −0.106	**0.035**
Males	−0.740 ± 0.801	−0.225	−0.925	−2.437, 0.957	0.369

*Note*: Boldface indicates significant *p* values at *p* < 0.05. Females, *N* = 13; Males, *N* = 18.

Abbreviations: B2, Baseline night 2; CI, confidence interval; KSS, Karolinska Sleepiness Scale; POMS‐F, Profile of Mood States Fatigue; SE, standard error; TSD, total sleep deprivation.

^a^ *N* = 17.

**TABLE 4:** Cognitive performance measures during total sleep deprivation (TSD) as significant predictors of recovery night 1 (R1) actigraphic sleep metrics.

TSD cognitive performance predictor and R1 sleep measure (*N* = 30)	Unstandardized *β* ± SE	Standardised *β*	*t*	95% CI	*p* (two‐tailed)
TSD DSST # correct and R1 sleep duration^a^	−2.029 ± 0.727	−0.473	−2.791	−3.521, −0.537	**0.010**
TSD DSST # correct and R1 SOL	0.194 ± 0.056	0.547	3.456	0.079, 0.310	**0.002**
TSD DSST # correct and R1 sleep onset^a^	0.017 ± 0.007	0.415	2.367	0.002, 0.033	**0.025**
TSD DS total # correct and R1 sleep onset^a^	0.048 ± 0.023	0.370	2.068	0.000, 0.096	**0.048**
TSD 10‐min. PVT lapses and R1 sleep duration^a^	2.842 ± 1.000	0.480	2.843	0.791, 4.893	**0.008**
TSD 10‐min. PVT lapses and R1 sleep efficiency	−0.331 ± 0.158	−0.369	−2.098	−0.654, −0.008	**0.045**
TSD 10‐min. PVT lapses and R1 WASO	2.767 ± 1.018	0.457	2.718	0.682, 4.853	**0.011**
TSD 10‐min. PVT lapses and R1 sleep percent	−0.363 ± 0.149	−0.417	−2.429	−0.669, −0.057	**0.022**
TSD 10‐min. PVT lapses and R1 sleep offset^a^	0.039 ± 0.013	0.489	2.911	0.011, 0.066	**0.007**

*Note*: Boldface indicates significant *p* values at *p* < 0.05.

Abbreviations: CI, confidence interval; DS, Digit Span Test; DSST, Digit Symbol Substitution Test; PVT, Psychomotor Vigilance Test; R1, recovery night 1; SE, standard error; SOL, sleep onset latency; TSD, total sleep deprivation; WASO, wake after sleep onset.

^a^ *N* = 29.

**TABLE 5:** Exploratory sex differences in cognitive performance measures during total sleep deprivation (TSD) as significant predictors of recovery night 1 (R1) actigraphic sleep metrics.

TSD cognitive performance predictor and R1 sleep measure	Unstandardized *β* ± SE	Standardised β	*t*	95% CI	*p* (two‐tailed)
TSD DSST # correct and R1 sleep duration					
Females	−2.518 ± 1.110	−0.583	−2.269	−4.992, −0.045	**0.047**
Males^a^	−1.195 ± 1.078	−0.275	−1.109	−3.493, 1.102	0.285
TSD DSST # correct and R1 SOL					
Females	0.064 ± 0.038	0.471	1.689	−0.020, 0.149	0.122
Males	0.359 ± 0.096	0.683	3.743	0.156, 0.562	**0.002**
TSD DSST # correct and R1 sleep onset					
Females	0.008 ± 0.012	0.208	0.673	−0.018, 0.033	0.516
Males^a^	0.034 ± 0.010	0.669	3.484	0.013, 0.054	**0.003**
TSD DS total # correct and R1 sleep onset					
Females	0.091 ± 0.034	0.642	2.646	0.014, 0.167	**0.024**
Males^a^	0.022 ± 0.031	0.178	0.702	−0.045, 0.089	0.493
TSD 10‐min. PVT lapses and R1 sleep duration					
Females	3.865 ± 1.151	0.728	3.359	1.301, 6.429	**0.007**
Males^a^	−2.169 ± 2.607	−0.210	−0.832	−7.726, 3.388	0.418
TSD 10‐min. PVT lapses and R1 sleep efficiency					
Females	−0.477 ± 0.167	−0.670	−2.854	−0.849, −0.105	**0.017**
Males	0.327 ± 0.450	0.179	0.726	−0.628, 1.281	0.478
TSD 10‐min. PVT lapses and R1 WASO					
Females	3.938 ± 1.084	0.754	3.634	1.523, 6.353	**0.005**
Males	−2.778 ± 2.724	−0.247	−1.020	−8.553, 2.996	0.323
TSD 10‐min. PVT lapses and R1 sleep percent					
Females	−0.537 ± 0.156	−0.735	−3.432	−0.885, −0.188	**0.006**
Males	0.407 ± 0.407	0.242	0.999	−0.457, 1.270	0.333
TSD 10‐min. PVT lapses and R1 sleep offset					
Females	0.057 ± 0.013	0.818	4.504	0.029, 0.085	**0.001**
Males^a^	−0.058 ± 0.034	−0.408	−1.729	−0.130, 0.014	0.104

*Note*: Boldface indicates significant *p* values at *p* < 0.05. Females, *N* = 12; Males, *N* = 18.

Abbreviations: CI, confidence interval; DS, Digit Span Test; DSST, Digit Symbol Substitution Test; PVT, Psychomotor Vigilance Test; R1, recovery night 1; SE, standard error; SOL, sleep onset latency; TSD, total sleep deprivation; WASO, wake after sleep onset.

^a^ *N* = 17.

**TABLE 6:** Pearson's correlation coefficients for baseline night 2 (B2) sleep measures.

Sleep measure	Sleep duration	Sleep onset	Sleep offset	Sleep midpoint	Sleep efficiency^a^	WASO	SOL^a^	Percent sleep
Sleep duration	—	−0.919**	0.166	−0.696**	0.031	0.255	−0.338	−0.109
Sleep onset	−0.919**	—	0.237	0.917**	0.014	−0.176	0.371*	0.038
Sleep offset	0.166	0.237	—	0.577**	0.091	0.186	0.035	−0.172
Sleep midpoint	−0.696**	0.917**	0.577**	—	−0.014	−0.008	0.282	−0.100
Sleep efficiency^a^	0.031	0.014	0.091	−0.014	—	−0.848**	−0.257	0.881**
WASO	0.255	−0.176	0.186	−0.008	−0.848**	—	−0.133	−0.988**
SOL^a^	−0.338	0.371*	0.035	0.282	−0.257	−0.133	—	0.096
Percent sleep	−0.109	0.038	−0.172	−0.100	0.881**	−0.988**	0.096	—

*Note*: **p < 0.01, *p < 0.05 (in bold); *N* = 31.

Abbreviations: SOL, sleep onset latency; WASO, wake after sleep onset.

^a^ *N* = 30.

**TABLE 7:** Pearson's correlation coefficients for recovery night 1 (R1) sleep measures.

Sleep measure	Sleep duration^a^	Sleep onset^a^	Sleep offset^a^	Sleep midpoint^a^	Sleep efficiency	WASO	SOL	Percent sleep
Sleep duration^a^	—	−0.592**	0.809**	0.225	−0.525**	0.688**	−0.175	−0.613**
Sleep onset^a^	−0.592**	—	−0.005	0.616**	0.087	−0.122	0.455*	0.076
Sleep offset^a^	0.809**	−0.005	—	0.729**	−0.588**	0.764**	0.117	−0.705**
Sleep midpoint^a^	0.225	0.616**	0.729**	—	−0.512**	0.608**	0.439*	−0.604**
Sleep efficiency	−0.525**	0.087	−0.588**	−0.512**	—	−0.945**	−0.297	0.973**
WASO	0.688**	−0.122	0.764**	0.608**	−0.945**	—	0.142	−0.989**
SOL	−0.175	0.455*	0.117	0.439*	−0.297	0.142	—	−0.215
Percent sleep	−0.613**	0.076	−0.705**	−0.604**	0.973**	−0.989**	−0.215	—

*Note*: **p < 0.01, *p < 0.05 (in bold); *N* = 30.

Abbreviations: SOL, sleep onset latency; WASO, wake after sleep onset.

^a^ *N* = 29.

Similarly, higher DS total # correct during TSD significantly predicted later R1 sleep onset (*r* = 0.370): each 1‐point increase in TSD DS total # correct significantly predicted a 0.048 h delay in R1 sleep onset (Table 4 and Figure 2D). TSD DS total # correct predicted 13.7% of the variance in R1 sleep onset. In addition, in females, each 1‐point increase in TSD DS total # correct significantly predicted a 0.091 h delay in R1 sleep onset (*r* = 0.642; *R* ^2^ = 0.412; Table 5).

**FIGURE 3:**
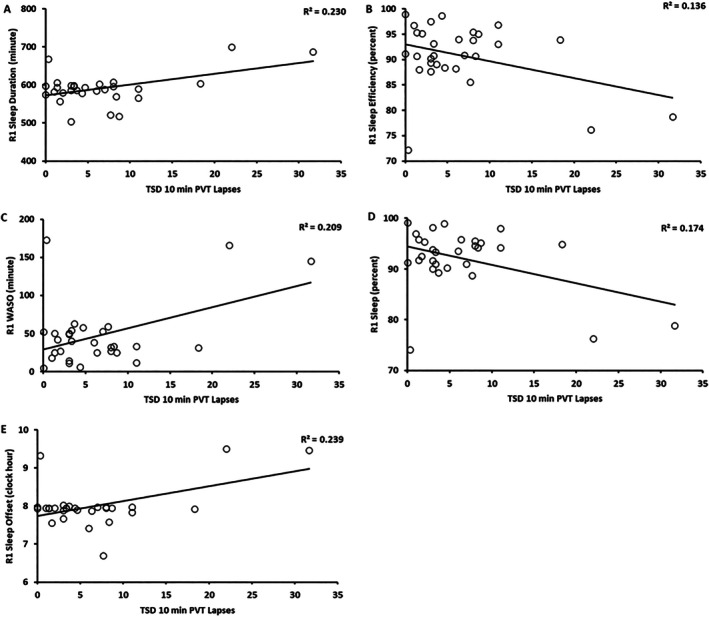
Scatter plots displaying behavioural attention measures during total sleep deprivation (TSD) as significant predictors of recovery night 1 (R1) actigraphic sleep measures. Simple linear regression found that a higher number of TSD 10‐min. Psychomotor Vigilance Test (PVT) lapses (reaction time > 500 ms) significantly predicted (A) longer R1 sleep duration, (B) lower R1 sleep efficiency, (C) higher R1 wake after sleep onset (WASO), (D) lower R1 percent sleep, and (E) later R1 sleep offset. The regression line and *R* ^2^ for each plot are shown. *p* < 0.05 was significant. *N* = 29 for (A) and (E). *N* = 30 for (B)–(D).

Furthermore, a higher number of 10‐min. PVT lapses during TSD (indicating poorer performance) significantly predicted longer R1 sleep duration, lower R1 sleep efficiency, higher R1 wake after sleep onset (WASO), lower R1 percent sleep, and later R1 sleep offset (*r*: −0.417‐0.489): each 1 lapse increase on the TSD 10‐min. PVT predicted a 2.842 min. increase in R1 sleep duration, a 0.331% decrease in R1 sleep efficiency, a 2.767 min. increase in R1 WASO, a 0.363% decrease in R1 percent sleep, and a 0.039 h delay in R1 sleep offset (Table 4 and Figure 3A–E). TSD PVT lapses predicted 13.6%–23.9% of the variance in R1 sleep duration, R1 sleep efficiency, R1 WASO, R1 percent sleep, and R1 sleep offset. Furthermore, in females, each one lapse increase on the TSD 10‐min. PVT predicted a 3.865 min. increase in R1 sleep duration (*r* = 0.728; *R* ^2^ = 0.530), a 0.477% decrease in R1 sleep efficiency (*r* = −0.670; *R* ^2^ = 0.449), a 3.938 min. increase in R1 WASO (*r* = 0.754; *R* ^2^ = 0.569), a 0.537% decrease in R1 percent sleep (*r* = −0.735; *R* ^2^ = 0.541), and a 0.057 h delay in R1 sleep offset (*r* = 0.818; *R* ^2^ = 0.670; Table 5).

There were no other significant cognitive performance measures during TSD as predictors of R1 actigraphic sleep measures, and there were no other significant predictors in females and males. In addition, age was not a significant factor.

### 3.3 Correlations Between B2 Sleep Metrics and Between R1 Sleep Metrics

Pearson's correlation analyses found significant correlations between B2 night sleep measures (Table 6) and between R1 night sleep measures (Table 7).

## 4 Discussion

Our findings demonstrated that novel bidirectional predictors exist between baseline and recovery sleep measures and neurobehavioural measures during TSD. We found greater B2 sleep efficiency predicted lower TSD KSS scores, and later B2 sleep onset and midpoint predicted lower TSD POMS‐F scores. Overall, B2 sleep measures predicted 14.2%–17.2% of the variance in TSD subjective sleepiness and fatigue measures. B2 sleep measures did not predict cognitive measures during TSD, but these measures predicted R1 sleep. Better TSD DSST performance predicted shorter R1 sleep duration, longer SOL, and later sleep onset; and better TSD DS performance predicted later sleep onset. Furthermore, greater TSD 10‐min PVT lapses (indicating poorer performance) predicted longer R1 sleep duration, lower sleep efficiency, greater WASO, lower percent sleep, and later sleep offset. Overall, cognitive performance measures during TSD predicted 13.6%–29.9% of the variance in R1 sleep measures. By contrast, TSD KSS and POMS‐F did not predict R1 sleep measures. Notably, females demonstrated more significant predictive bidirectional relationships. In summary, we demonstrate that baseline sleep predicts resilience to subjective sleepiness and fatigue during TSD, whereas cognitive performance resilience during TSD predicts sleep during recovery.

We found that greater B2 sleep efficiency predicted lower TSD KSS scores, and later B2 sleep onset and midpoint predicted lower TSD POMS‐F scores. KSS and POMS‐F metrics are distinguishable from each other (Casale et al. 2022; Bermudez et al. 2016; Franzen et al. 2008); this is reflected in our KSS results, which may be due to the dissipation of Process S, and in our fatigue results, which may be due to sleep timing or circadian factors. Our B2 sleep results explained 14.2%–17.2% of the variance, which is on par with one study that found locus coeruleus microstructural integrity predicted individual differences in neurobehavioural responses to sleep deprivation (Quan et al. 2024). In addition, a recent study found that hypothalamus–dorsal striatum connectivity predicted individual differences in sleepiness without sleep deprivation (Mao et al. 2024). Other factors that might predict sleepiness include REM sleep length, N1 sleep variation, and N1 ratio (Mao et al. 2023). By contrast, B2 sleep measures failed to predict DS, DSST, or PVT performance, which is in line with studies showing subjective measures are distinct from objective performance during sleep loss (Yamazaki and Goel 2020; Tkachenko and Dinges 2018; Dennis et al. 2017). Notably, our results are consistent with a study without sleep loss (Leng et al. 2024), but in contrast to other research with and without sleep restriction (Swanson et al. 2021; Neylan et al. 2010; Galli et al. 2022).

We also found that better TSD DSST performance predicted shorter R1 sleep duration, greater SOL, and later sleep onset; and better TSD DS performance predicted later R1 sleep onset. Therefore, if an individual is more resilient during TSD, as reflected in better performance on the DS and DSST, this is reflected by shorter recovery sleep duration. In addition, our findings showed greater TSD 10‐min. PVT lapses predicted longer R1 sleep duration, lower sleep efficiency, greater WASO, lower percent sleep, and later sleep offset. Thus, if an individual is less resilient during TSD, as reflected in poorer performance on the PVT, this is indicated by longer sleep duration and later sleep offset, with less efficiency and less percent sleep, and more WASO (fragmented sleep) during recovery. In almost all cases, the cognitive measures (cognitive throughput, working memory, and behavioural attention) during TSD predicted different R1 sleep outcomes, which may be due to the lack of correlation of these measures during sleep loss (Dennis et al. 2017), indicating they represent different domains of cognitive performance. Of note, subjective sleepiness and fatigue during TSD did not predict recovery sleep measures, in line with the fact that these subjective measures are distinct from objective performance. Even though recovery sleep is critical for neurobehavioural measures to return to baseline levels following TSD (Yamazaki, Antler, Lasek, et al. 2021), beyond this study, we know little about the factors that predict recovery sleep after TSD. Our research suggests it may be possible to intervene on neurobehavioural performance during TSD to improve subsequent recovery sleep; hence, future research should further investigate this important area of research.

Pearson's correlation analyses revealed significant relationships between B2 sleep metrics and between R1 sleep metrics. B2 sleep onset and sleep midpoint were positively associated, mirrored in their similar predictive relationships with TSD POMS‐F scores. Notably, B2 sleep duration, which was negatively associated with B2 sleep onset and B2 sleep midpoint, demonstrated a positive predictive relationship with TSD POMS‐F scores, but this did not reach statistical significance (*p* = 0.095). Our results also found that R1 sleep onset was negatively associated with R1 sleep duration and positively associated with R1 SOL, which reflects their predictive relationships with TSD DSST and DS scores. Of note, R1 sleep offset was positively associated with R1 sleep duration and R1 WASO, and negatively associated with R1 sleep efficiency and R1 percent sleep, as represented in their predictive relationships with TSD PVT lapses. Further investigation of these relationships is needed to enhance the biomarker knowledge base in this area.

Prior research has found sex differences in neurobehavioural measures (Åkerstedt et al. 2017; Santhi et al. 2016) as well as in sleep measures (Lok et al. 2024; Wright et al. 2023). In our study, most predictive bidirectional relationships between sleep metrics and TSD neurobehavioural measures remained significant and explained more of the variance in females. Of note, this is the first examination of bidirectional relationships between baseline and recovery sleep and neurobehavioural measures during TSD in females and males. Future research should investigate these bidirectional relationships using larger samples.

Although these studies were conducted in an isolation facility, our results have possible implications for individuals who are sleep deprived due to work obligations or lifestyle choices and who exhibit phenotypic vulnerability to the neurobehavioural deficits caused by sleep deprivation. Examining these bidirectional predictive relationships could assist in recommending mitigation strategies in relation to real‐world settings including medical services, transportation, spaceflight, and the military, as well as other domains (Dawson et al. 2021; Wada et al. 2024; Cosgrave et al. 2018; Stahn et al. 2023). This research may also be useful for mathematical models predicting neurobehavioural metrics during sleep loss (Fu and Ma 2023; Cochrane et al. 2021). Furthermore, we assume that the significant predictive bidirectional relationships between these indices would be maintained after repeated exposures to TSD since we have previously demonstrated that these measures are robustly stable across time during sleep loss (Dennis et al. 2017; Yamazaki and Goel 2020; Pasetes and Goel 2024). Notably, targeting B2 sleep may modify and improve neurobehavioural responses to TSD, and targeting neurobehavioural responses during TSD may achieve better recovery sleep.

Our studies had a few limitations. Due to our small sample size, we did not correct for multiple comparisons for our main findings; as such, future studies should verify the results of this study using larger sample sizes. Further, while we recognise that both circadian and homeostatic sleep pressure components influence neurobehavioural measures at different times of day, we did not analyse each time point separately since our time points were not evenly distributed throughout the day (e.g., every 2 h). In addition, although sleep midpoint was used as a proxy of circadian phase, we did not use any other measures of circadian phase. Notably, we also could not control for or investigate menstrual cycle or menstrual phase in these studies. We did not use polysomnography (PSG) in this study; thus, the possibility of microsleeps occurring during TSD cannot be ruled out. Although actigraphic sleep measures have been shown to have comparable stability to PSG across pre‐experimental, baseline, and recovery sleep phases surrounding TSD in other studies (Pasetes and Goel 2024), actigraphic sleep efficiency may be less accurate compared to PSG. Furthermore, it is difficult to generalise our results to populations with mood or sleep disorders, or to those with other medical conditions because our participants were healthy and thoroughly screened prior to participation.

## 5 Conclusion

Our novel findings demonstrate that baseline sleep predicts subjective sleepiness and fatigue resilience during TSD, whereas cognitive performance resilience during TSD predicts subsequent recovery sleep measures. Thus, sleep efficiency, sleep onset, and sleep midpoint during fully rested conditions may serve as biomarkers to predict individual differences in sleepiness and fatigue measures during TSD, and PVT lapses, DSST # correct, and DS total # correct during TSD may be biomarkers for determining individual differences in recovery sleep. Identifying sleep and cognitive biomarkers for these individual differences may provide a better understanding of underlying mechanisms and may be useful for developing personalised strategies for managing fatigue and sleepiness, and sleep–wake recovery, respectively. In addition, almost all the predictive bidirectional relationships between sleep metrics and neurobehavioural measures remained significant and explained more of the variance in females. In summary, our results underscore predictors, mechanisms, and biomarkers between sleep health and individual differences in neurobehavioural performance during sleep loss.

## Author Contributions

**Lauren N. Pasetes:** validation, formal analysis, writing – original draft, writing – review and editing, visualization. **Namni Goel:** conceptualization, methodology, validation, investigation, resources, data curation, writing – original draft, writing – review and editing, visualization, supervision, project administration, funding acquisition.

## Conflicts of Interest

The authors declare no conflicts of interest.

## Data Availability

The data generated during and/or analysed during the current study are available from the corresponding author on reasonable request.

## References

[jsr70031-bib-0001] Åkerstedt, T. , A. Anund , J. Axelsson , and G. Kecklund . 2014. “Subjective Sleepiness Is a Sensitive Indicator of Insufficient Sleep and Impaired Waking Function.” Journal of Sleep Research 23: 242–254. 10.1111/jsr.12158. 24750198

[jsr70031-bib-0002] Åkerstedt, T. , and M. Gillberg . 1990. “Subjective and Objective Sleepiness in the Active Individual.” International Journal of Neuroscience 52: 29–37. 10.3109/00207459008994241. 2265922

[jsr70031-bib-0003] Åkerstedt, T. , D. Hallvig , and G. Kecklund . 2017. “Normative Data on the Diurnal Pattern of the Karolinska Sleepiness Scale Ratings and Its Relation to Age, Sex, Work, Stress, Sleep Quality and Sickness Absence/Illness in a Large Sample of Daytime Workers.” Journal of Sleep Research 26: 559–566. 10.1111/jsr.12528. 28370590

[jsr70031-bib-0004] Basner, M. , and D. F. Dinges . 2011. “Maximizing Sensitivity of the Psychomotor Vigilance Test (PVT) to Sleep Loss.” Sleep 34: 581–591. 10.1093/sleep/34.5.581. 21532951 PMC3079937

[jsr70031-bib-0005] Bermudez, E. B. , E. B. Klerman , C. A. Czeisler , D. A. Cohen , J. K. Wyatt , and A. J. Phillips . 2016. “Prediction of Vigilant Attention and Cognitive Performance Using Self‐Reported Alertness, Circadian Phase, Hours Since Awakening, and Accumulated Sleep Loss.” PLoS One 11: e0151770. 10.1371/journal.pone.0151770. 27019198 PMC4809494

[jsr70031-bib-0006] Blackburn, H. L. , and A. L. Benton . 1957. “Revised Administration and Scoring of the Digit Span Test.” Journal of Consulting Psychology 21: 139–143. 10.1037/h0047235. 13416432

[jsr70031-bib-0007] Bourgeois, A. , A. LeUnes , and M. Meyers . 2010. “Full‐Scale and Short‐Form of the Profile of Mood States: A Factor Analytic Comparison.” Journal of Sport Behavior 33: 355–376.

[jsr70031-bib-0008] Brieva, T. E. , C. E. Casale , E. M. Yamazaki , C. A. Antler , and N. Goel . 2021. “Cognitive Throughput and Working Memory Raw Scores Consistently Differentiate Resilient and Vulnerable Groups to Sleep Loss.” Sleep 44: zsab197. 10.1093/sleep/zsab197. 34333658 PMC8664585

[jsr70031-bib-0009] Casale, C. E. , E. M. Yamazaki , T. E. Brieva , C. A. Antler , and N. Goel . 2022. “Raw Scores on Subjective Sleepiness, Fatigue, and Vigor Metrics Consistently Define Resilience and Vulnerability to Sleep Loss.” Sleep 45: zsab228. 10.1093/sleep/zsab228. 34499166 PMC8754490

[jsr70031-bib-0010] Cochrane, C. , D. Ba , E. B. Klerman , and M. A. St. Hilaire . 2021. “An Ensemble Mixed Effects Model of Sleep Loss and Performance.” Journal of Theoretical Biology 509: 110497. 10.1016/j.jtbi.2020.110497. 32966825 PMC8631086

[jsr70031-bib-0011] Cosgrave, J. , L. J. Wu , M. van den Berg , T. L. Signal , and P. H. Gander . 2018. “Sleep on Long Haul Layovers and Pilot Fatigue at the Start of the Next Duty Period.” Aerospace Medicine and Human Performance 89: 19–25. 10.3357/AMHP.4965.2018. 29233240

[jsr70031-bib-0012] Dawson, D. , M. Sprajcer , and M. Thomas . 2021. “How Much Sleep Do You Need? A Comprehensive Review of Fatigue Related Impairment and the Capacity to Work or Drive Safely.” Accident Analysis and Prevention 151: 105955. 10.1016/j.aap.2020.105955. 33383522

[jsr70031-bib-0013] Dennis, L. E. , R. J. Wohl , L. A. Selame , and N. Goel . 2017. “Healthy Adults Display Long‐Term Trait‐Like Neurobehavioral Resilience and Vulnerability to Sleep Loss.” Scientific Reports 7: 14889. 10.1038/s41598-017-14006-7. 29097703 PMC5668275

[jsr70031-bib-0015] Fonseca, L. M. , M. G. Finlay , N. S. Chaytor , et al. 2024. “Mid‐Life Sleep Is Associated With Cognitive Performance Later in Life in Aging American Indians: Data From the Strong Heart Study.” Frontiers in Aging Neuroscience 16: 1346807. 10.3389/fnagi.2024.1346807. 38903901 PMC11188442

[jsr70031-bib-0016] Franzen, P. L. , G. J. Siegle , and D. J. Buysse . 2008. “Relationships Between Affect, Vigilance, and Sleepiness Following Sleep Deprivation.” Journal of Sleep Research 17: 34–41. 10.1111/j.1365-2869.2008.00635.x. 18275553 PMC3107826

[jsr70031-bib-0017] Fu, J. , and L. Ma . 2023. “Individualisation Method of Biomathematical Model of Fatigue for Predicting Individual Performance in Mild and Irregular Sleep Deprivation.” Ergonomics 66: 1310–1324. 10.1080/00140139.2022.2146206. 36369843

[jsr70031-bib-0018] Galli, O. , C. W. Jones , O. Larson , M. Basner , and D. F. Dinges . 2022. “Predictors of Interindividual Differences in Vulnerability to Neurobehavioral Consequences of Chronic Partial Sleep Restriction.” Sleep 45: zsab278. 10.1093/sleep/zsab278. 34897501 PMC8754493

[jsr70031-bib-0019] Gilmore, G. R. , A. L. Smith , F. B. Dickinson , A. D. Crosswell , W. B. Mendes , and L. N. Whitehurst . 2024. “Sleep/Wake Regularity Influences How Stress Shapes Executive Function.” Frontiers in Sleep 3: 1359723. 10.3389/frsle.2024.1359723. PMC12713908 41424511

[jsr70031-bib-0020] Goel, N. , M. Basner , H. Rao , and D. F. Dinges . 2013. “Circadian Rhythms, Sleep Deprivation, and Human Performance.” Progress in Molecular Biology and Translational Science 119: 155–190. 10.1016/B978-0-12-396971-2.00007-5. 23899598 PMC3963479

[jsr70031-bib-0021] Honn, K. A. , T. Halverson , M. L. Jackson , et al. 2020. “New Insights Into the Cognitive Effects of Sleep Deprivation by Decomposition of a Cognitive Throughput Task.” Sleep 43: zsz319. 10.1093/sleep/zsz319. 32227081 PMC7355397

[jsr70031-bib-0022] Jones, C. W. , M. Basner , D. J. Mollicone , C. M. Mott , and D. F. Dinges . 2022. “Sleep Deficiency in Spaceflight Is Associated With Degraded Neurobehavioral Functions and Elevated Stress in Astronauts on Six‐Month Missions Aboard the International Space Station.” Sleep 45: zsac006. 10.1093/sleep/zsac006. 35023565 PMC8919197

[jsr70031-bib-0023] Killgore, W. D. , N. L. Grugle , R. M. Reichardt , D. B. Killgore , and T. J. Balkin . 2009. “Executive Functions and the Ability to Sustain Vigilance During Sleep Loss.” Aviation, Space, and Environmental Medicine 80: 81–87. 10.3357/asem.2396.2009. 19198192

[jsr70031-bib-0024] Leng, Y. , K. Knutson , M. R. Carnethon , and K. Yaffe . 2024. “Association Between Sleep Quantity and Quality in Early Adulthood With Cognitive Function in Midlife.” Neurology 102: e208056. 10.1212/WNL.0000000000208056. 38170947 PMC10870739

[jsr70031-bib-0025] Lim, J. , and D. F. Dinges . 2010. “A Meta‐Analysis of the Impact of Short‐Term Sleep Deprivation on Cognitive Variables.” Psychological Bulletin 136: 375–389. 10.1037/a0018883. 20438143 PMC3290659

[jsr70031-bib-0026] Lok, R. , J. Qian , and S. L. Chellappa . 2024. “Sex Differences in Sleep, Circadian Rhythms, and Metabolism: Implications for Precision Medicine.” Sleep Medicine Reviews 75: 101926. 10.1016/j.smrv.2024.101926. 38564856

[jsr70031-bib-0027] Mao, T. , Y. Chai , B. Guo , P. Quan , and H. Rao . 2023. “Sleep Architecture and Sleep EEG Alterations Are Associated With Impaired Cognition Under Sleep Restriction.” Nature and Science of Sleep 15: 823–838. 10.2147/NSS.S420650. PMC10578164 37850195

[jsr70031-bib-0028] Mao, T. , B. Guo , P. Quan , et al. 2024. “Morning Resting Hypothalamus‐Dorsal Striatum Connectivity Predicts Individual Differences in Diurnal Sleepiness Accumulation.” NeuroImage 299: 120833. 10.1016/j.neuroimage.2024.120833. 39233125

[jsr70031-bib-0029] Mathew, G. M. , S. M. Strayer , D. S. Bailey , et al. 2021. “Changes in Subjective Motivation and Effort During Sleep Restriction Moderate Interindividual Differences in Attentional Performance in Healthy Young Men.” Nature and Science of Sleep 13: 1117–1136. 10.2147/NSS.S294409. PMC8286723 34285617

[jsr70031-bib-0030] McNair, D. , M. Lorr , and L. Droppleman . 1971. Manual for the Profile of Mood States. Educational and Industrial Testing Services.

[jsr70031-bib-0031] Moreno‐Villanueva, M. , G. von Scheven , A. Feiveson , A. Bürkle , H. Wu , and N. Goel . 2018. “The Degree of Radiation‐Induced DNA Strand Breaks Is Altered by Acute Sleep Deprivation and Psychological Stress and Is Associated With Cognitive Performance in Humans.” Sleep 41: 1–9. 10.1093/sleep/zsy067. 29596659

[jsr70031-bib-0032] Neylan, T. C. , T. J. Metzler , C. Henn‐Haase , et al. 2010. “Prior Night Sleep Duration Is Associated With Psychomotor Vigilance in a Healthy Sample of Police Academy Recruits.” Chronobiology International 27: 1493–1508. 10.3109/07420528.2010.504992. 20795888 PMC13003608

[jsr70031-bib-0033] Pasetes, L. N. , and N. Goel . 2024. “Short‐Term and Long‐Term Phenotypic Stability of Actigraphic Sleep Metrics Involving Repeated Sleep Loss and Recovery.” Journal of Sleep Research 33: e14149. 10.1111/jsr.14149. 38284151 PMC11284248

[jsr70031-bib-0034] Pasetes, L. N. , K. M. Rosendahl‐Garcia , and N. Goel . 2023a. “Impact of Bimonthly Repeated Total Sleep Deprivation and Recovery Sleep on Cardiovascular Indices.” Physiological Reports 11: e15841. 10.14814/phy2.15841. 37849046 PMC10582224

[jsr70031-bib-0035] Pasetes, L. N. , K. M. Rosendahl‐Garcia , and N. Goel . 2023b. “Cardiovascular Measures Display Robust Phenotypic Stability Across Long‐Duration Intervals Involving Repeated Sleep Deprivation and Recovery.” Frontiers in Neuroscience 17: 1201637. 10.3389/fnins.2023.1201637. 37547137 PMC10397520

[jsr70031-bib-0036] Quan, P. , T. Mao , X. Zhang , et al. 2024. “Locus Coeruleus Microstructural Integrity Is Associated With Vigilance Vulnerability to Sleep Deprivation.” Human Brain Mapping 45: e70013. 10.1002/hbm.70013. 39225144 PMC11369684

[jsr70031-bib-0037] Santhi, N. , A. S. Lazar , P. J. McCabe , J. C. Lo , J. A. Groeger , and D. J. Dijk . 2016. “Sex Differences in the Circadian Regulation of Sleep and Waking Cognition in Humans.” Proceedings of the National Academy of Sciences of the United States of America 113: E2730–E2739. 10.1073/pnas.1521637113. 27091961 PMC4868418

[jsr70031-bib-0038] Spaeth, A. M. , N. Goel , and D. F. Dinges . 2012. “Managing Neurobehavioral Capability When Social Expediency Trumps Biological Imperatives.” Progress in Brain Research 199: 377–398. 10.1016/B978-0-444-59427-3.00021-6. 22877676 PMC3600847

[jsr70031-bib-0039] Stahn, A. C. , D. Bucher , P. Zu Eulenburg , et al. 2023. “Paving the Way to Better Understand the Effects of Prolonged Spaceflight on Operational Performance and Its Neural Bases.” Npj Microgravity 9: 59. 10.1038/s41526-023-00295-y. 37524737 PMC10390562

[jsr70031-bib-0040] Swanson, L. M. , M. M. Hood , M. H. Hall , et al. 2021. “Associations Between Sleep and Cognitive Performance in a Racially/Ethnically Diverse Cohort: The Study of Women's Health Across the Nation.” Sleep 44: zsaa182. 10.1093/sleep/zsaa182. 32918472 PMC7879413

[jsr70031-bib-0041] Tkachenko, O. , and D. F. Dinges . 2018. “Interindividual Variability in Neurobehavioral Response to Sleep Loss: A Comprehensive Review.” Neuroscience and Biobehavioral Reviews 89: 29–48. 10.1016/j.neubiorev.2018.03.017. 29563066

[jsr70031-bib-0014] Van Dongen, H. P. , M. D. Baynard , G. Maislin , and D. F. Dinges . 2004. “Systematic Interindividual Differences in Neurobehavioral Impairment From Sleep Loss: Evidence of Trait‐Like Differential Vulnerability.” Sleep 27: 423–433. 10.1093/sleep/27.3.423. 15164894

[jsr70031-bib-0042] Wada, H. , M. Basner , M. Cordoza , D. Dinges , and T. Tanigawa . 2024. “Objective Alertness, Rather Than Sleep Duration, Is Associated With Burnout and Depression: A National Survey of Japanese Physicians.” Journal of Sleep Research 34: e14304. 10.1111/jsr.14304. 39134926 PMC11745938

[jsr70031-bib-0043] Wechsler, D. 1981. WAIS‐R Manual: Wechsler Adult Intelligence Scale Revised. Psychological Corporation.

[jsr70031-bib-0044] Wingelaar‐Jagt, Y. Q. , T. T. Wingelaar , W. J. Riedel , and J. G. Ramaekers . 2024. “Comparison of Effects of Modafinil and Caffeine on Fatigue‐Vulnerable and Fatigue‐Resistant Aircrew After a Limited Period of Sleep Deprivation.” Frontiers in Physiology 14: 1303758. 10.3389/fphys.2023.1303758. 38260091 PMC10800817

[jsr70031-bib-0045] Wright, C. J. , S. Milosavljevic , and A. Pocivavsek . 2023. “The Stress of Losing Sleep: Sex‐Specific Neurobiological Outcomes.” Neurobiology of Stress 24: 100543. 10.1016/j.ynstr.2023.100543. 37252645 PMC10209346

[jsr70031-bib-0046] Yamazaki, E. M. , C. A. Antler , C. E. Casale , L. E. MacMullen , A. J. Ecker , and N. Goel . 2021. “Cortisol and C‐Reactive Protein Vary During Sleep Loss and Recovery but Are Not Markers of Neurobehavioral Resilience.” Frontiers in Physiology 12: 782860. 10.3389/fphys.2021.782860. 34912243 PMC8667577

[jsr70031-bib-0047] Yamazaki, E. M. , C. A. Antler , C. R. Lasek , and N. Goel . 2021. “Residual, Differential Neurobehavioral Deficits Linger After Multiple Recovery Nights Following Chronic Sleep Restriction or Acute Total Sleep Deprivation.” Sleep 44: zsaa224. 10.1093/sleep/zsaa224. 33274389 PMC8274462

[jsr70031-bib-0048] Yamazaki, E. M. , C. E. Casale , T. E. Brieva , C. A. Antler , and N. Goel . 2022. “Concordance of Multiple Methods to Define Resiliency and Vulnerability to Sleep Loss Depends on Psychomotor Vigilance Test Metric.” Sleep 45: zsab249. 10.1093/sleep/zsab249. 34624897 PMC8754491

[jsr70031-bib-0049] Yamazaki, E. M. , and N. Goel . 2020. “Robust Stability of Trait‐Like Vulnerability or Resilience to Common Types of Sleep Deprivation in a Large Sample of Adults.” Sleep 43: zsz292. 10.1093/sleep/zsz292. 31784748 PMC8152927

[jsr70031-bib-0050] Yamazaki, E. M. , K. M. Rosendahl‐Garcia , C. E. Casale , et al. 2022. “Left Ventricular Ejection Time Measured by Echocardiography Differentiates Neurobehavioral Resilience and Vulnerability to Sleep Loss and Stress.” Frontiers in Physiology 12: 795321. 10.3389/fphys.2021.795321. 35087419 PMC8787291

